# In Memoriam—Frederick R. Campbell, M. D.

**Published:** 1888-10

**Authors:** 


BUFFALO MEDICAL AND SURGICAL JOURNAL
A MONTHLY REVIEW OF MEDICINE AND SURGERY.

EDITORS:
THOS. LOTHROP, M. D., - - - W. W. POTTER, M. D.

All communications, whether of a literary or business character, should be addressed to the editors: 284 FRANKLIN STREET, BUFFALO, N. Y.

### Editorial.

### IN MEMORIAM.

### FREDERICK R. CAMPBELL, M. D.

Died Sept. 14, 1888, aged 28 years.

We convey to our numerous readers the intelligence ot another sorrow that has befallen the editorial corps of the JOURNAL in the death of Dr. Campbell, which took place at Rochester, whither he had gone for needed rest. He fell a victim to typhoid fever, after a sickness of over a month.

The history of Dr. Campbell's life is one of the deepest interest, in that it shows the reward of unremitting toil and application. He was born in Niagara county, this State, in 1860. His parents were Scotch. Graduating at the Lockport Union School at the early age of sixteen years, he entered Rochester University, and completed his freshman year in that institution· The sophomore and junior studies were completed in one year at the University of the City of New York. Returning to Rochester, he entered the University, and finished his collegiate course, receiving the degree of Bachelor of Arts, and subsequently that of M. A. in course. He received his medical degree from the Buffalo Medical College, and the *ad eundem* degree of Doctor in Medicine from Niagara University in 1884.

In 1883, he became connected with the Medical Department of Niagara University, first as assistant in Surgery, then as Lecturer on Hygiene, and subsequently as Professor of Materia Medica and Therapeutics. For several years he was connected with the Board of Health as Sanitary Inspector and District Health Physician. His connection with this journal as associate editor is known to our readers in the various contributions from his pen which have appeared in our numbers.

It will be estimated that only the most industrious habits of life could have enabled him to have accomplished so much at his early age. Besides a large and increasing private practice, Dr. Campbell found the time to gather the materials for the work recently published, "Language of Medicine," which demonstrated to the profession that, as a writer, he had few equals in our city in any department to which he devoted his energies.

It may be said that, as a lecturer on hygiene, and then on materia medica, he amassed a wealth of knowledge which surprised his colleagues, and enabled him to hold the attention of the class, commanding the respect of all by the ability and earnestness which he brought to the various positions which he occupied.

Dr. Campbell earned the respect of the profession in this city by the uprightness of his life, and the erudition he displayed as a physician, journalist, and medical teacher. So much could only have been attained by the closest study. Indeed, it may be said that his death was the result of overwork, enfeebling a physical system not robust, and making it an easy prey to disease.

We lament deeply this sad event. The community and the profession suffer an irreparable loss in the early taking off of a life so full of promise. Few attain, at his early age, the position and the professional influence which came to him as the reward of honest and earnest work. The future, also, was bright in the expectation of promotion which his superior ability and attainments commanded.

At a special meeting of the Faculty of the Medical Department of Niagara University, the following memorial was adopted:

Frederick R. Campbell, M.D., died at Rochester, N. Y., September 14, 1888, at the age of twenty-eight years. In his death, this Faculty is bereft of another esteemed and beloved associate, and the affliction comes even while our eyes are still filled with tears, and our hearts heavy with grief, from the loss of Dr. A. R. Davidson.

Another man of usefulness and worth, and full of great promise to the community, to the medical profession, and to this College, has been called to rest. Although he was still in the morning of life, and his work had but just begun, yet he had already won a large measure of distinction as a medical teacher, practitioner, sanitarian, and writer, and the result of his labors will stand as a lasting monument to his industry and ripe scholarship.

As an associate with this Faculty, first as assistant to the late Dr. C. C. F. Gay, then as Lecturer on Hygiene, and lastly as Professor of Materia Medica and Therapeutics, he was valued for his integrity, faithfulness, diligence, and learning, and as a teacher he proved himself able, clear, and impressive.

We part with him with deepest sorrow and grief, but humbly bow to the will of the All-Wise.

His bereaved companion and friends have our sincere sympathy.

---

The Buffalo Obstetrical Society, at its regular meeting, September 25, 1888, took appropriate action, as follows:

For the second time in the history of this society, we are called upon to mourn the death of one of our fellows. And while it is proper to have some formal record of our sorrow, it is most difficult to find words to sufficiently express it. Dr. Campbell was a most valued member of this society, as he was of any organization that was honored with his presence. He contributed from time to time to its advancement, and many an intricate question raised here was made plainer by his learning. He had a breadth of vision that few men, even of greater age, can boast. He was ever a student, liberal and modest. It seemed strange to him that where so much is unknown—by far the greater part—that man could feel proud of the little knowledge he possesses. It was a pleasure to see with what simplicity he received praise for his work. As notice after notice of his book came to him, he studied them carefully, first to find what there was in criticism, afterward to appreciate what there was of encouragement. Seldom has it been our fortune to know a more simple character.

It is sad when the old die, but what is there to be said when one so young and full of promise is taken away? Let us learn the lesson of his short life. Let us devote ourselves to honest labor for the advancement of our profession, and when our time comes to be

> "Delivered from the galling yoke of time
> And these frail elements,"

may his example have taught us how to obtain the reward we feel sure awaited him as he passed "To where beyond these voices there is peace."

